# Development of a Novel Soft Tissue Measurement Device for Individualized Finite Element Modeling in Custom-Fit CPAP Mask Evaluation

**DOI:** 10.1007/s10439-024-03581-2

**Published:** 2024-07-08

**Authors:** Erica Martelly, Summer Lee, Kristina Martinez, Sandeep Rana, Kenji Shimada

**Affiliations:** 1 Department of Mechanical Engineering, Carnegie Mellon University, 5000 Forbes Avenue, Pittsburgh, PA 15213 USA; 2 Department of Neurology, Allegheny General Hospital, 490 E North Avenue, Pittsburgh, PA 15212 USA

**Keywords:** Elastic modulus, Material properties, Soft tissue, Face, Indenter

## Abstract

**Purpose:**

Individual facial soft tissue properties are necessary for creating individualized finite element (FE) models to evaluate medical devices such as continuous positive airway pressure (CPAP) masks. There are no standard tools available to measure facial soft tissue elastic moduli, and techniques in literature require advanced equipment or custom parts to replicate.

**Methods:**

We propose a simple and inexpensive soft tissue measurement (STM) indenter device to estimate facial soft tissue elasticity at five sites: chin, cheek near lip, below cheekbone, cheekbone, and cheek. The STM device consists of a probe with a linear actuator and force sensor, an adjustment system for probe orientation, a head support frame, and a controller. The device was validated on six ballistics gel samples and then tested on 28 subjects. Soft tissue thickness was also collected for each subject using ultrasound.

**Results:**

Thickness and elastic modulus measurements were successfully collected for all subjects. The mean elastic modulus for each site is *E*_c_ = 53.04 ± 20.97 kPa for the chin, *E*_l_ = 16.33 ± 8.37 kPa for the cheek near lip, *E*_bc_ = 27.09 ± 11.38 kPa for below cheekbone, *E*_cb_ = 64.79 ± 17.12 kPa for the cheekbone, and *E*_ch_ = 16.20 ± 5.09 kPa for the cheek. The thickness and elastic modulus values are in the range of previously reported values. One subject’s measured soft tissue elastic moduli and thickness were used to evaluate custom-fit CPAP mask fit in comparison to a model of that subject with arbitrary elastic moduli and thickness. The model with measured values more closely resembles in vivo leakage results.

**Conclusion:**

Overall, the STM provides a first estimate of facial soft tissue elasticity and is affordable and easy to build with mostly off-the-shelf parts. These values can be used to create personalized FE models to evaluate custom-fit CPAP masks.

## Introduction

Human facial traits vary widely, with statistically significant ethnic, racial, gender, and weight differences in facial traits [1–3]. This poses a challenge when modeling human faces in simulation for various applications such as surgical planning or evaluation of medical devices. To produce a finite element (FE) model that accurately represents each unique human face, we need not only their facial contour, but also their facial skin thicknesses and elastic moduli at various sites. Including these individual properties in simulation ensures that the predictions made in simulation are accurate. Ultrasound is a well-researched technique used to measure soft tissue thickness, but there are no commercially available tools or methodologies for measuring the modulus of elasticity of soft tissue, especially in vivo.

The problem of measuring the elastic modulus of soft tissue in vivo has been tackled by various other research groups on different parts of the body. Many have used indenters [4–9], while others have used suction [10, 11] or a microrobot [12]. Some devices collect the elasticity of multiple layers of soft tissue [4–6, 10], while others focus on the external layers only [7–9, 11, 12]. All the methods mentioned here were able to successfully measure the modulus of elasticity of skin at various locations including facial tissue [8–10, 12], limbs [5, 6, 11], organs [7], and the breast, buttocks, and umbilicus regions [4]. However, all the methods used were unique and required specialized equipment or precision manufactured parts to function. None used a commercially available product to take their measurements. This makes it difficult for other researchers to replicate their techniques to collect their own soft tissue elastic moduli.

In this paper, we present an inexpensive and easy to build indenter device to provide a first estimate of facial soft tissue elasticity at five testing sites. In this case, we intend to use these estimates for a facial FE model evaluating the fit of our previously designed custom-fit CPAP mask [13]. We show an example of a FE simulation to evaluate custom-fit CPAP mask fit for one subject using their soft tissue thickness and elasticity. However, the device can work on more regions of the face and can be adapted to other parts of the body. The soft tissue measurement (STM) device is inexpensive, simple to build from mostly off-the-shelf parts, and can easily be replicated by other researchers for their own soft material measurements.

## Materials and Methods

### Indentation Theory

The fundamental principle that this device is based on is that the elastic modulus can be obtained using indentation. This method is described by McKee et al., which overviews the use of various indenters to obtain the elastic modulus of soft biological tissues [14]. By using this equation, we are assuming soft tissue is a homogeneous linear elastic material, which it is not, so the elasticity measured will be an estimate. For the indentation method, the equation used depends on the type of indenter. We are using a cylindrical indenter which leads us to use Eq. 1 where *F* is the force applied by the indenter, *ν* is the Poisson’s ratio of the material being tested, *R* is the indenter radius, and *δ* is the indentation depth. The indenter has a 7.5 mm radius, the force and indentation depth are collected by our device, and the Poisson’s ratio used is from literature. We use a Poisson’s ratio of 0.49 for human tissue [15].

1 $$E= \frac{F(1-{\nu }^{2})}{2R\delta }.$$

### Device Description

**Fig. 1:**
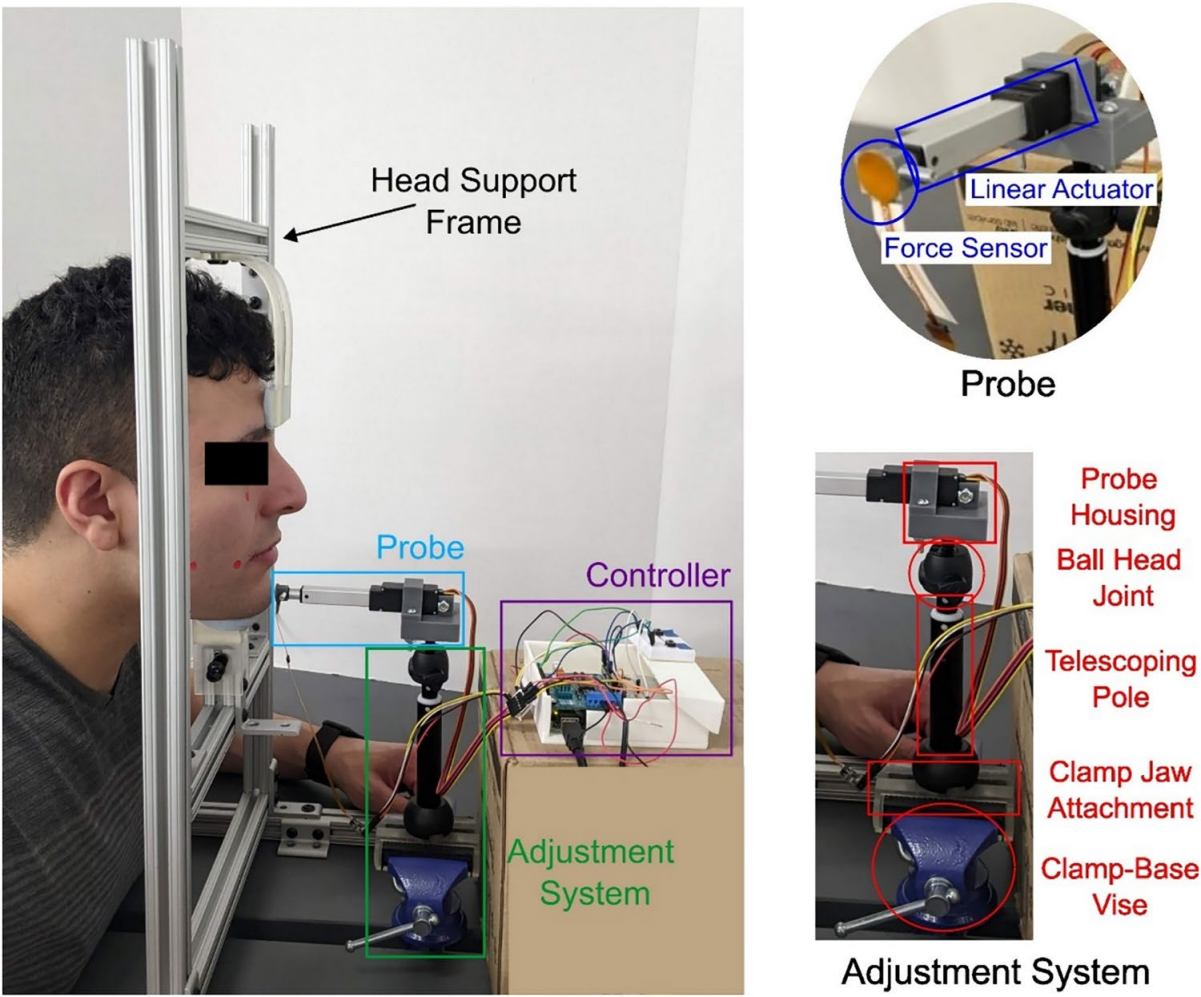
Complete STM device while testing on a subject, showing the four parts: head support frame, probe, adjustment system, and controller. The probe and adjustment system are shown in more detail

**Fig. 2:**
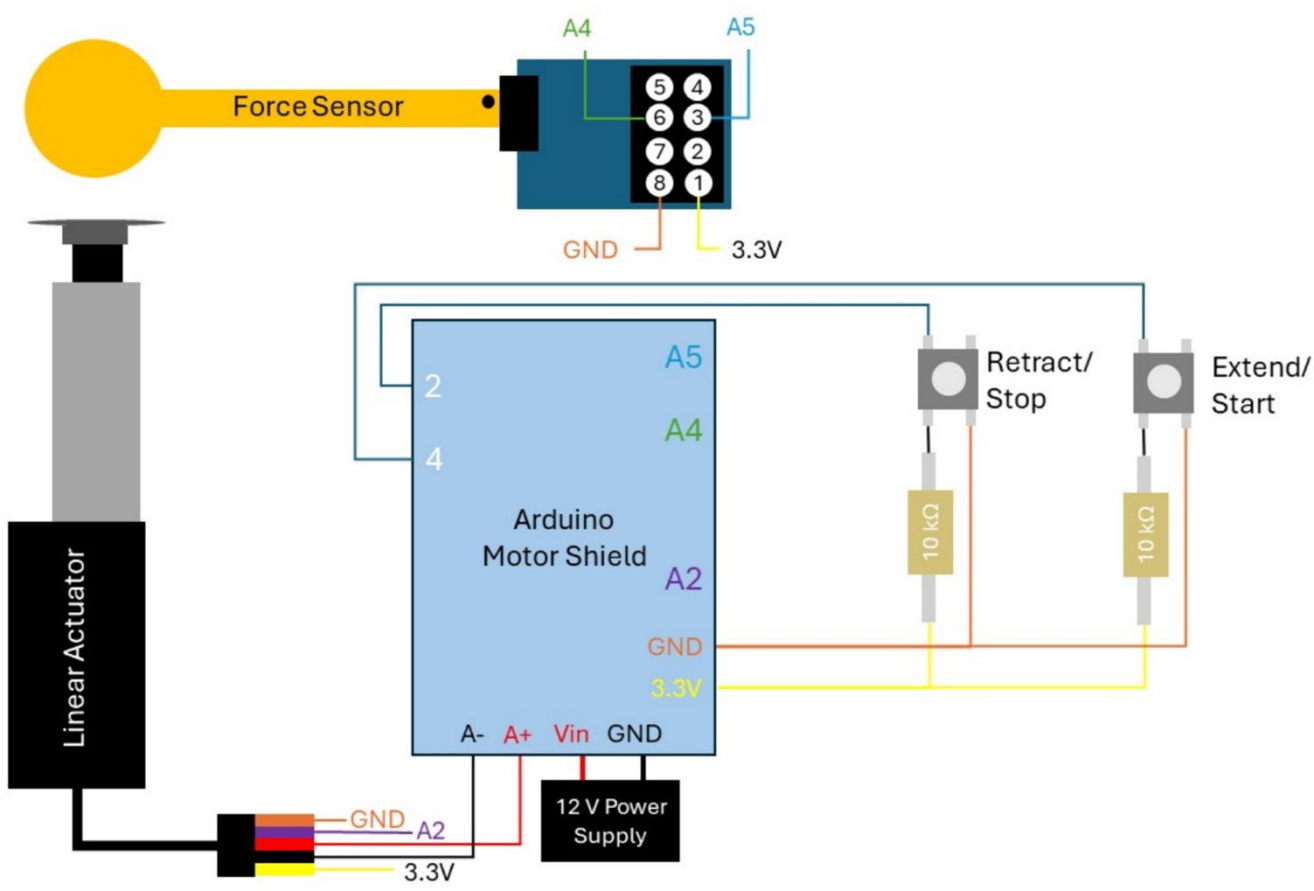
Schematic of STM controller. The buttons are used to extend and retract the probe when finding the initial displacement, and to start or stop the test during the automated elasticity testing

The soft tissue measurement (STM) device shown in Fig. 1 consists of four parts: the head support frame, the probe, the adjustment system, and the controller. The head support frame is a metal frame with silicone cushions to support the subject’s chin and forehead during testing. This frame helps the subject remain stationary during testing and provides resistance to the probe. The probe consists of an L16-P 35:1 linear actuator with a 50 mm stroke (Actuonix Motion Devices, Inc., Saanichton, Canada), a 15 mm diameter 3D-printed circular indenter attachment, and a calibrated 15 mm diameter force sensor with an I2C board (SingleTact, PPS UK Limited, Glasgow, UK). The linear actuator is powered by a 12 V DC power supply. The force sensor can measure force up to 45 N with a force resolution of 0.09 N and is taped to a 3D-printed attachment that connects to the tip of the actuator. The probe is attached to the adjustment system using an additively manufactured housing which is then glued to the ball joint head. The adjustment system consists of a ball-head joint on a telescoping pole, taken from a small camera tripod, which is attached to a clamp-base vise using 3D-printed clamp jaw attachments. This system allows the probe to be adjusted to various positions on the face. The electronics are controlled by an Arduino MEGA, an Arduino motor shield, and two buttons, which control the actuator and collect the displacement and force data from the actuator and force sensor, respectively. A schematic for the controller is shown in Fig. 2. The device is made of mostly off-the-shelf parts, except for five custom pieces, all additively manufactured on a Formlabs Form 2 stereolithography printer (Formlabs, Somerville, MA): a forehead bar, a chin rest, a force sensor attachment to the actuator, a mounting attachment for the ball-head joint, and two clamp jaw attachments.

### Elasticity Testing Methodology

**Fig. 3:**
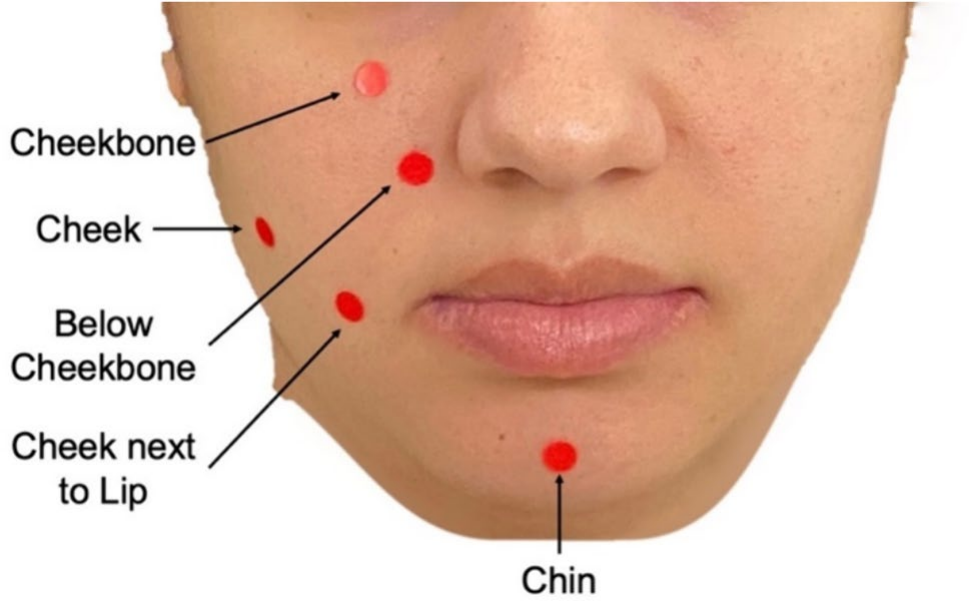
The five facial soft tissue testing sites shown on a face with red markers

Approval for testing on human subjects was obtained from the Institutional Review Board of Carnegie Mellon University (STUDY2023_00000154). The procedures used in this study adhere to the tenets of the Declaration of Helsinki. All subjects tested consented to participation in the study. For elasticity testing on human subjects, we start by marking five testing locations on the subject’s face, all on the right side, as shown in Fig. 3. The subject is asked to place their chin and forehead on the head support frame and the probe is manually adjusted to touch the surface of the skin perpendicular to the face at the site being tested by moving the adjustment system. The actuator is manually extended using the two buttons (one to extend, one to retract) to just touch the surface of the face and the initial displacement is recorded. Once the initial displacement is recorded, an automated program runs the test. The actuator drives the circular attachment and the force sensor in toward the face. The actuator then stops driving forward when it experiences a decrease in velocity. This is calculated by taking the displacement of the actuator in half-second intervals. If the change in displacement is less than 0.15 mm, a threshold set during the validation of the device, the actuator will stop. While it is stopped, the Arduino MEGA collects one value for the force experienced by the force sensor and one value for the depth of indentation, which is given by the actuator. This process is automatically repeated five times at the same site. The entire process is repeated for each of the five testing locations. The force and displacement values can then be used to find the elastic modulus of the soft tissue on the face.

### Device Validation

**Fig. 4:**
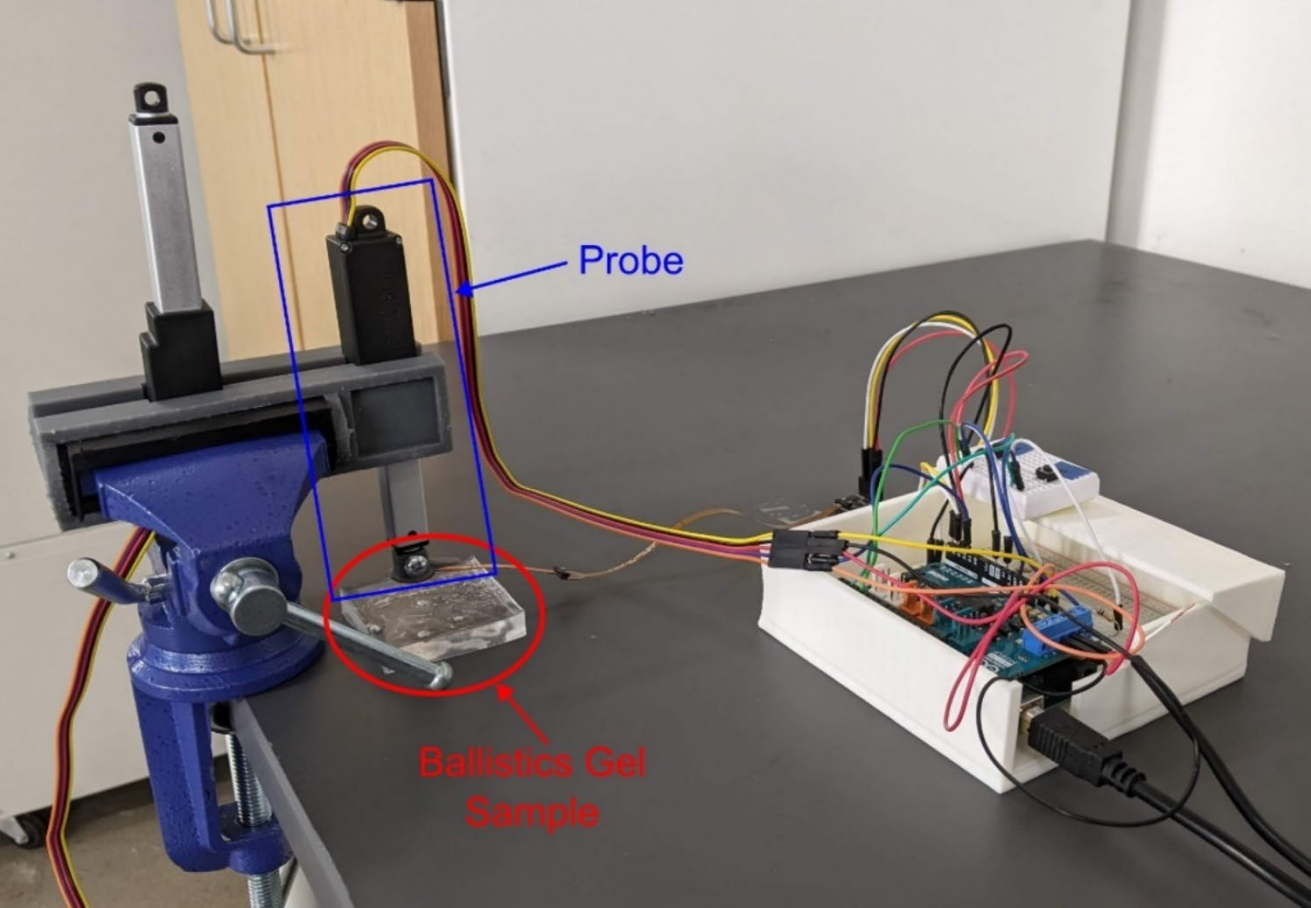
Validation testing setup on ballistics gel sample where the probe is oriented down toward the table where the sample rests

**Fig. 5:**
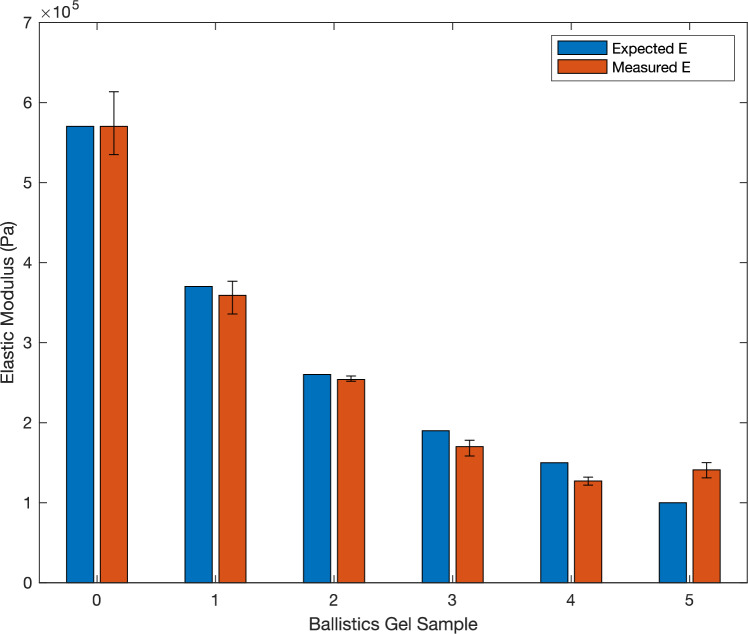
Bar chart of elastic modulus (*E*) with error bars for the ballistics gel samples as measured by the STM compared to the expected *E* reported by the manufacturer

Before testing the device on human subjects, we tested the device on ballistics gel samples to validate its performance and understand the error. Six ballistics gel samples were obtained from Humimic Medical (Greenville, SC, USA). This type of ballistics gel is made to mimic human tissue. These six samples were melted into approximately 50 mm × 50 mm × 9.5 mm rectangular prisms for testing. The validation setup is slightly different from the testing setup for human subjects. For validation, instead of having the probe on a ball head, it is attached to the clamp-base vise facing down using a 3D-printed clamp as shown in Fig. 4. The sample is then placed on the table below the probe as shown in Fig. 4. The initial displacement from the end of the circular attachment to the sample is calculated by extending the actuator toward the sample until a force of 1 N is read by the force sensor. The displacement is recorded and then the actuator retracts. Each of the six ballistics gel samples was tested 3 times, with 5 data points collected per trial. The results are summarized in Table 1 and Fig. 5. The measured elastic modulus correlates well with the reported elasticity, with all measured values being the correct order of magnitude compared to the reported elasticity. The error was low for Samples 0 to 2, with an error below 3%. The softer samples (Samples 3 to 5) resulted in a higher error, with an especially high error of 41% for Sample 5.

**Table 1:** Validation testing results with ballistics gel samples

Ballistics gel sample	Expected *E* (Pa)	Measured *E* (Pa)	Error (%)
0	5.70 × 10^5^	5.70 × 10^5^ ± 3.98 × 10^4^	0.06
1	3.70 × 10^5^	3.59 × 10^5^ ± 2.10 × 10^4^	2.97
2	2.70 × 10^5^	2.54 × 10^5^ ± 3.64 × 10^3^	2.29
3	1.90 × 10^5^	1.70 × 10^5^ ± 1.04 × 10^4^	10.61
4	1.50 × 10^5^	1.27 × 10^5^ ± 5.04 × 10^3^	15.54
5	1.00 × 10^5^	1.41 × 10^5^ ± 9.67 × 10^3^	41.49

### Thickness Testing Methodology

**Fig. 6:**
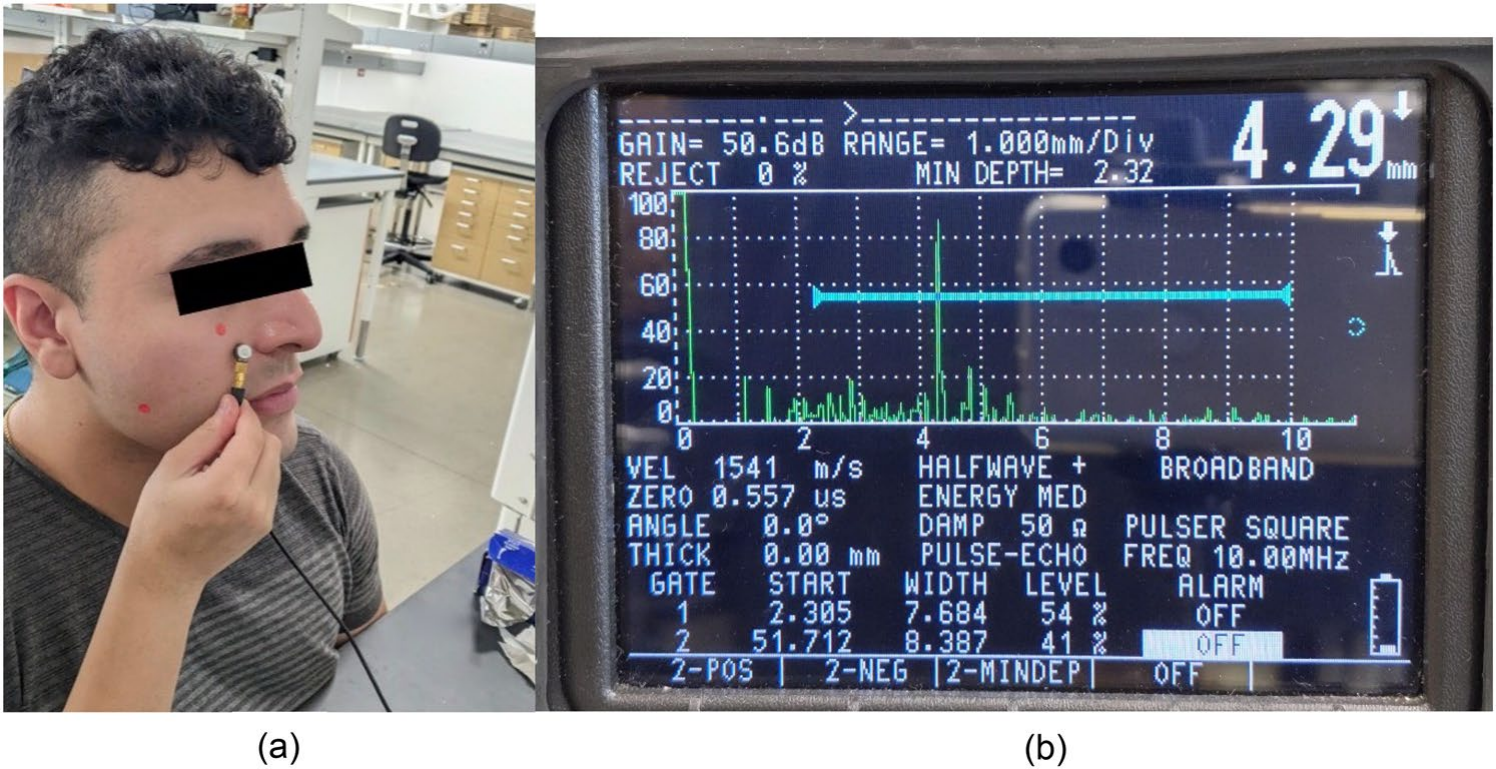
Ultrasound testing on a subject (**a**) and example result on ultrasound display (**b**)

Soft tissue thickness was measured using ultrasound, a well-researched method for collecting tissue thickness. The device and technique used were adapted from De Greef et al. [16]. Like De Greef et al., we used an Epoch 4 Plus ultrasound A-mode scanner (Panametrics, Billerica, MA, USA) along with a quarter-inch diameter cylindrical 10 MHz transducer. The small size of the transducer allowed us to collect soft tissue thickness at a localized region of the face. The speed of sound through human soft tissue is cited as 1540 m/s [17], and this value was set on our device. We used a camera in front of the device output screen to record readings. Figure 6a shows the probe being used on a subject’s face, while Fig. 6b shows an example of the ultrasound result. Each site was measured three times. The probe is applied to the face with light pressure to avoid compressing the tissue and affecting the measurement. The average of three measurements was used as the result.

## Results

The STM device and ultrasound were tested on 28 subjects using the method described in Sect. "Materials and Methods". Overall, there were 14 female and 14 male subjects, aged 19–54 with an average age of 26.4 ± 8.5 and an average body mass index (BMI) of 24.3 ± 3.31. One subject self-identified as non-binary and was assigned to the male group due to their sex assigned at birth. The subject racial demographics are 50% Asian, 14% Black or African American, 29% White and 7% mixed race. Five subjects (18%) also identified as Hispanic or Latino.

### Elasticity Results

**Fig. 7:**
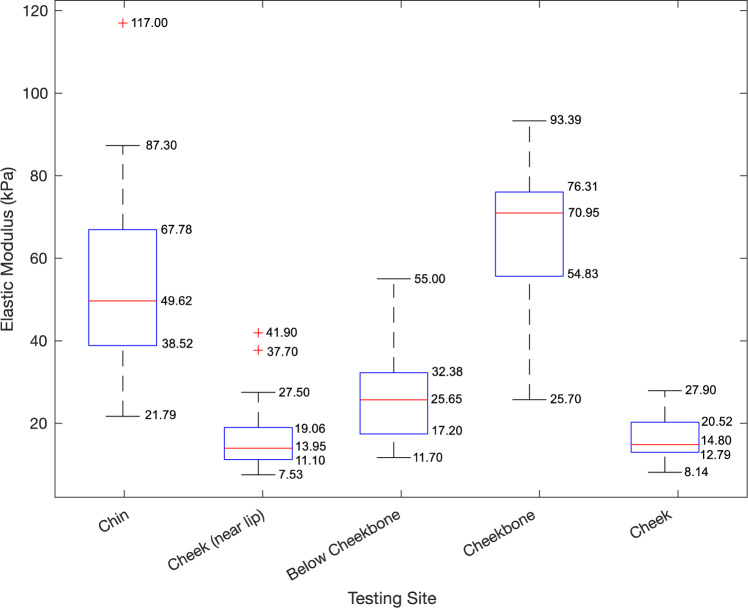
Box and whisker plot with mean, minimum, maximum, and outliers of elasticity measured for each site (in kPa)

The elastic moduli of the five facial sites measured with the STM device are summarized in Fig. 7 and detailed in Table 2. The age, BMI, and sex of each of the 28 subjects are also detailed in Table 2. The mean elastic modulus for each site is *E*_c_ = 53.04 ± 20.97 kPa for the chin, *E*_l_ = 16.33 ± 8.37 kPa for the cheek near lip, *E*_bc_ = 27.09 ± 11.38 kPa for below cheekbone, *E*_cb_ = 64.79 ± 17.12 kPa for the cheekbone, and *E*_ch_ = 16.20 ± 5.09 kPa for the cheek.

**Table 2:** Summary of elasticity testing results including age, sex (M for male, F for female), body mass index (BMI), measured elastic modulus *E* (in kPa), and standard deviation (SD) for each subject (i) at each testing site

Subject #/sex	Age (years)	BMI (kg/m^2^)	Chin *E*_c_ ± SD (kPa)	Cheek near lip *E*_l_ ± SD (kPa)	Below cheekbone *E*_bc_ ± SD (kPa)	Cheekbone *E*_cb_ ± SD (kPa)	Cheek *E*_ch_ ± SD (kPa)
1/M	23	29.3	30.60 ± 1.64	14.30 ± 0.46	37.90 ± 1.36	71.10 ± 6.02	21.60 ± 1.38
2/F	20	22.3	65.30 ± 3.15	10.50 ± 0.87	32.00 ± 1.92	71.60 ± 3.80	18.10 ± 0.62
3/M	24	24.1	21.70 ± 2.64	12.60 ± 1.43	14.00 ± 0.77	38.10 ± 2.20	8.14 ± 0.96
4/F	27	21.9	32.80 ± 2.35	11.40 ± 2.49	14.20 ± 0.82	34.20 ± 2.56	13.60 ± 0.88
5/M	20	19.2	23.70 ± 2.53	12.60 ± 1.04	25.90 ± 0.96	60.50 ± 7.12	11.40 ± 0.83
6/F	23	24.6	54.50 ± 3.47	8.07 ± 1.03	11.70 ± 0.83	25.70 ± 3.33	14.90 ± 1.19
7/F	19	22.8	35.10 ± 5.21	7.53 ± 1.14	32.50 ± 12.80	76.60 ± 11.40	9.98 ± 1.29
8/F	19	23.5	74.70 ± 5.10	21.50 ± 3.53	30.40 ± 3.16	39.60 ± 3.11	13.40 ± 0.98
9/F	20	21.3	55.70 ± 4.87	8.61 ± 0.63	35.10 ± 5.46	54.00 ± 6.96	14.50 ± 2.90
10/M	23	22.0	43.70 ± 3.14	11.00 ± 0.91	17.80 ± 1.89	80.20 ± 6.23	18.50 ± 0.68
11/F	32	28.6	43.50 ± 3.59	14.40 ± 1.66	12.30 ± 1.43	72.30 ± 6.28	8.42 ± 1.03
12/F	26	20.7	50.80 ± 5.30	15.20 ± 2.71	17.00 ± 1.76	66.70 ± 8.29	14.50 ± 1.37
13/F	26	21.3	48.00 ± 8.40	13.90 ± 2.60	23.70 ± 3.15	57.30 ± 6.50	12.10 ± 1.54
14/F	29	26.1	78.80 ± 8.00	8.43 ± 0.93	14.30 ± 0.62	64.40 ± 2.52	14.80 ± 2.01
15/M	20	20.0	39.50 ± 5.39	14.00 ± 0.91	12.50 ± 0.40	57.40 ± 5.07	19.70 ± 3.08
16/M	21	23.4	43.00 ± 2.53	27.50 ± 8.62	39.30 ± 2.66	78.40 ± 5.09	8.73 ± 0.41
17/F	26	22.0	31.70 ± 3.31	41.90 ± 6.54	55.00 ± 8.44	93.30 ± 16.60	19.40 ± 1.73
18/M	28	29.7	117.00 ± 13.40	22.70 ± 2.41	51.10 ± 7.08	83.30 ± 5.54	14.70 ± 1.35
19/M	28	23.5	87.30 ± 5.68	25.70 ± 2.17	31.70 ± 3.95	70.80 ± 2.46	26.00 ± 2.74
20/M	26	23.3	54.00 ± 5.17	37.70 ± 3.82	21.90 ± 3.08	85.90 ± 9.45	27.90 ± 4.26
21/F	24	25.4	46.10 ± 2.56	19.10 ± 4.17	25.40 ± 1.90	71.60 ± 9.44	14.80 ± 1.01
22/F	54	26.0	68.60 ± 5.04	13.30 ± 1.78	23.90 ± 2.61	59.10 ± 5.15	21.30 ± 2.70
23/M	54	25.6	49.50 ± 7.70	11.80 ± 1.30	42.90 ± 3.40	74.40 ± 6.45	14.00 ± 1.94
24/M	24	34.7	38.19 ± 2.26	14.55 ± 0.79	28.12 ± 0.42	41.34 ± 1.02	20.79 ± 1.42
25/F	27	24.2	57.29 ± 5.31	10.43 ± 1.97	30.64 ± 2.46	51.69 ± 2.73	20.84 ± 3.92
26/M	24	28.1	71.83 ± 12.20	12.53 ± 0.96	21.30 ± 2.03	75.45 ± 13.90	12.58 ± 1.79
27/M	29	23.0	49.74 ± 2.48	17.17 ± 0.70	25.30 ± 1.73	73.68 ± 7.25	17.24 ± 1.75
28/M	24	24.2	72.61 ± 6.11	18.94 ± 1.46	30.61 ± 2.97	85.48 ± 10.50	21.70 ± 2.81
Mean ± SD	26.4 ± 8.5	24.3 ± 3.31	53.04 ± 20.97	16.33 ± 8.37	27.09 ± 11.38	64.79 ± 17.12	16.20 ± 5.09

Two-sample paired *t* tests were performed between the results at each testing site. The *p* values for these tests are summarized in Table 3. From these tests, we see that the difference between all sites is statistically significant except for the cheek near lip (*E*_l_) and the cheek (*E*_ch_) (*p* = 0.943). There is no correlation between stiffness and age or BMI. Between genders, men appear to have a slightly higher average stiffness for all sites except the chin, but there is no statistical difference between the measurements. The groups were also separated by BMI with 19 subjects with normal BMI (19–24.9) and 9 subjects with overweight or obese BMI (25+). There were no statistical differences between the elasticities in each BMI group. Regardless, there is wide variation from subject to subject, as evidenced by the large standard deviation of the mean at each site.

**Table 3:** *P* values for *t* tests between each site

	Cheek near lip	Below cheekbone	Cheekbone	Cheek
Chin	3.713E−10	8.867E−07	0.026	4.601E−10
Cheek near lip		0.00019	3.098E−16	0.943
Below cheekbone			8.339E−13	4.499E−05
Cheekbone				1.576E−15

### Thickness Results

**Fig. 8:**
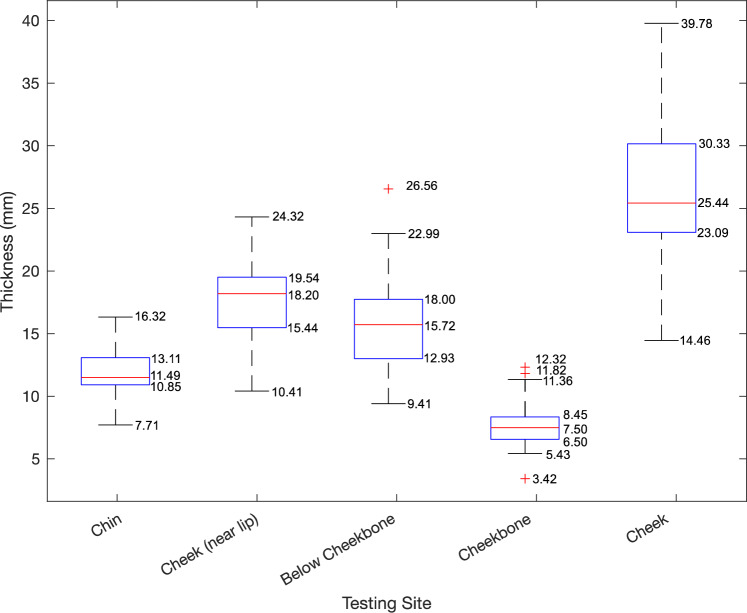
Box and whisker plot with mean, minimum, maximum and outliers of thickness measured for each site (in mm)

The facial soft tissue thicknesses measured at each site are summarized in Fig. 8 and detailed in Table 4 along with sex, age, and BMI. The mean thickness for each site is *T*_c_ = 11.97 ± 2.08 mm for the chin, *T*_l_ = 17.63 ± 3.07 mm for the cheek near lip, *T*_bc_ = 16.17 ± 4.22 mm for below cheekbone, *T*_cb_ = 7.76 ± 2.02 mm for the cheekbone, and *T*_ch_ = 26.67 ± 5.51 mm for the cheek.

**Table 4:** Summary of thickness testing results including age, sex (M for male, F for female), body mass index (BMI), measured elastic modulus *E* (in kPa), and standard deviation (SD) for each subject (i) at each testing site

Subject #/sex	Age (years)	BMI (kg/m^2^)	Chin *T*_c_ ± SD (kPa)	Cheek near lip *T*_l_ ± SD (kPa)	Below cheekbone *T*_bc_ ± SD (kPa)	Cheekbone *T*_cb_ ± SD (kPa)	Cheek *T*_ch_ ± SD (kPa)
1/M	23	29.3	11.52 ± 0.44	17.57 ± 0.14	16.77 ± 0.57	5.50 ± 0.30	29.84 ± 1.37
2/F	20	22.3	10.60 ± 0.41	15.57 ± 1.05	21.91 ± 0.48	7.87 ± 0.16	23.91 ± 0.64
3/M	24	24.1	11.58 ± 1.62	15.31 ± 1.11	12.86 ± 1.52	5.43 ± 0.34	25.20 ± 0.33
4/F	27	21.9	11.08 ± 0.28	19.31 ± 0.80	9.41 ± 0.22	12.32 ± 0.91	28.07 ± 0.56
5/M	20	19.2	11.05 ± 0.70	13.28 ± 0.06	11.13 ± 0.72	6.85 ± 0.38	34.40 ± 0.35
6/F	23	24.6	10.35 ± 0.13	14.27 ± 0.46	22.62 ± 1.14	11.82 ± 0.87	32.98 ± 0.43
7/F	19	22.8	13.14 ± 0.66	17.70 ± 1.18	17.26 ± 0.44	7.46 ± 0.34	25.41 ± 1.01
8/F	19	23.5	11.42 ± 0.30	18.99 ± 0.63	13.15 ± 0.62	11.36 ± 1.13	30.49 ± 0.33
9/F	20	21.3	9.86 ± 0.19	20.05 ± 1.56	15.71 ± 1.14	7.64 ± 0.24	25.25 ± 1.09
10/M	23	22.0	11.70 ± 0.27	15.92 ± 0.2	15.69 ± 0.38	8.16 ± 0.51	14.46 ± 0.32
11/F	32	28.6	15.06 ± 0.17	19.02 ± 0.49	15.36 ± 0.75	7.27 ± 0.47	21.58 ± 0.79
12/F	26	20.7	9.38 ± 0.18	14.57 ± 0.35	13.34 ± 0.35	9.88 ± 0.40	29.66 ± 0.50
13/F	26	21.3	10.78 ± 0.39	15.39 ± 0.17	11.55 ± 0.23	9.99 ± 0.25	22.74 ± 0.46
14/F	29	26.1	16.32 ± 0.33	19.19 ± 0.57	12.74 ± 0.46	6.98 ± 0.58	32.72 ± 0.19
15/M	20	20.0	12.84 ± 0.34	19.87 ± 0.42	11.99 ± 0.27	7.54 ± 0.68	24.36 ± 0.10
16/M	21	23.4	11.18 ± 0.34	19.42 ± 0.21	16.98 ± 0.07	7.16 ± 0.11	27.17 ± 0.65
17/F	26	22.0	16.10 ± 0.32	18.69 ± 0.25	15.72 ± 0.64	8.54 ± 0.22	23.08 ± 0.39
18/M	28	29.7	11.46 ± 0.13	19.77 ± 0.35	13.32 ± 0.29	7.73 ± 0.21	28.25 ± 0.20
19/M	28	23.5	12.97 ± 0.20	17.44 ± 0.71	16.71 ± 0.27	6.21 ± 0.14	25.46 ± 0.38
20/M	26	23.3	13.67 ± 0.27	16.34 ± 0.26	11.19 ± 0.35	6.68 ± 0.28	19.89 ± 0.08
21/F	24	25.4	12.13 ± 0.33	12.84 ± 0.09	16.74 ± 0.53	6.44 ± 0.16	25.18 ± 0.40
22/F	54	26.0	15.51 ± 0.24	22.85 ± 0.26	20.56 ± 0.34	10.05 ± 0.16	34.96 ± 0.59
23/M	54	25.6	13.01 ± 0.57	20.87 ± 0.82	17.10 ± 0.63	7.90 ± 0.90	27.59 ± 0.77
24/M	24	34.7	13.43 ± 0.26	19.58 ± 0.69	22.99 ± 1.70	8.02 ± 0.37	39.78 ± 0.59
25/F	27	24.2	11.28 ± 0.07	24.32 ± 0.57	26.56 ± 0.68	7.27 ± 0.07	23.10 ± 1.04
26/M	24	28.1	7.71 ± 0.19	19.25 ± 0.49	21.80 ± 0.70	6.25 ± 0.12	31.60 ± 1.34
27/M	29	23.0	11.04 ± 0.29	10.41 ± 0.36	18.24 ± 0.42	5.44 ± 0.22	19.90 ± 0.37
28/M	24	24.2	8.94 ± 0.19	15.82 ± 0.07	13.29 ± 0.72	3.42 ± 0.24	19.75 ± 0.39
Mean ± SD	26.4 ± 8.5	24.3	11.97 ± 2.08	17.63 ± 3.07	16.17 ± 4.22	7.76 ± 2.02	26.67 ± 5.51

### Correlation Between Elasticity and Thickness

The Pearson correlation coefficient was calculated between the elasticity and thickness at each testing site. Only one site, the cheekbone, showed a strong negative correlation between elasticity and thickness (*r* =  − 0.54), and this correlation was statistically significant (*p* = 0.003). For this sample, the cheekbone elasticity increases as the thickness decreases. The average elasticity and thickness showed a strong negative correlation (*r* =  − 0.87), which fits the intuition that thicker sites have lower stiffness. However, due to only having five testing sites, the correlation is not statistically significant (*p* = 0.052). Separating the data by sex, the female group showed a significant negative correlation between elasticity and thickness at the cheekbone (*r* =  − 0.7, *p* = 0.005), while the male group had a negligible correlation (*r* =  − 0.12) for that site. No correlations were found between elasticity and age or BMI.

### Applications of Measured Elasticity and Thickness in FE Analysis

In previous work, we developed an FE model to assess the fit of our custom-fit CPAP masks and included a skull modified using free form deformation to define the soft tissue thickness at the cheekbone and chin. In that work, we used arbitrary values for cheekbone and chin soft tissue thickness based on literature values [18]. We wanted to create an FE model with subjects measured soft tissue thicknesses and elasticities to improve model fidelity. Now that we have validated the use of ultrasound for soft tissue thickness measurements and developed the STM device for facial soft tissue elasticity measurements, we can incorporate some of these measurements into the FE model. Subject 1 from the prior FE work was used in this analysis, this is not the same Subject 1 reported earlier.

**Fig. 9:**
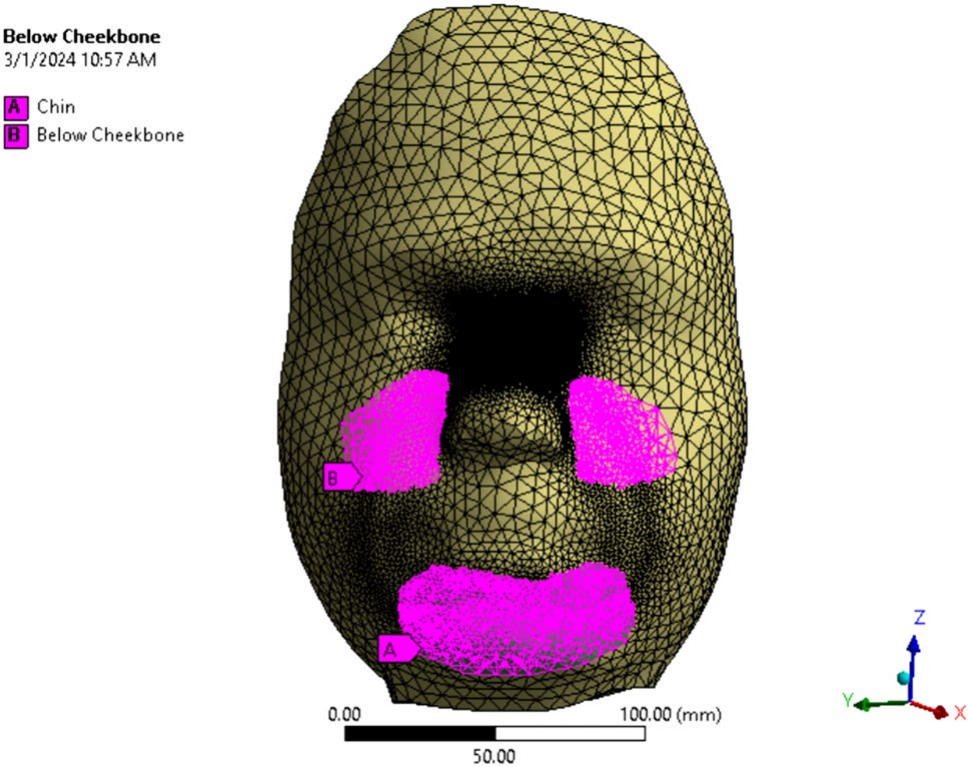
Face in finite element software showing the chin and below cheekbone regions where the measured soft tissue elasticity was assigned

We measured the soft tissue thickness and elasticity at various sites for Subject 1. The chin and cheekbone thicknesses were collected and measured as 12.65 ± 0.24 mm for the chin and 6.41 ± 0.39 mm for the cheekbone. The facial soft tissue elasticity was measured as 65.64 ± 6.02 kPa for the chin, 27.05 ± 3.40 kPa for below cheekbone, and 14.76 ± 2.68 kPa for the cheek. The values for the chin and cheekbone thicknesses were used to modify Subject 1’s skull. Figure 9 shows the chin and below cheekbone regions where the corresponding measured elasticity was defined. The rest of the face was assigned the elasticity of the cheek.

**Fig. 10:**
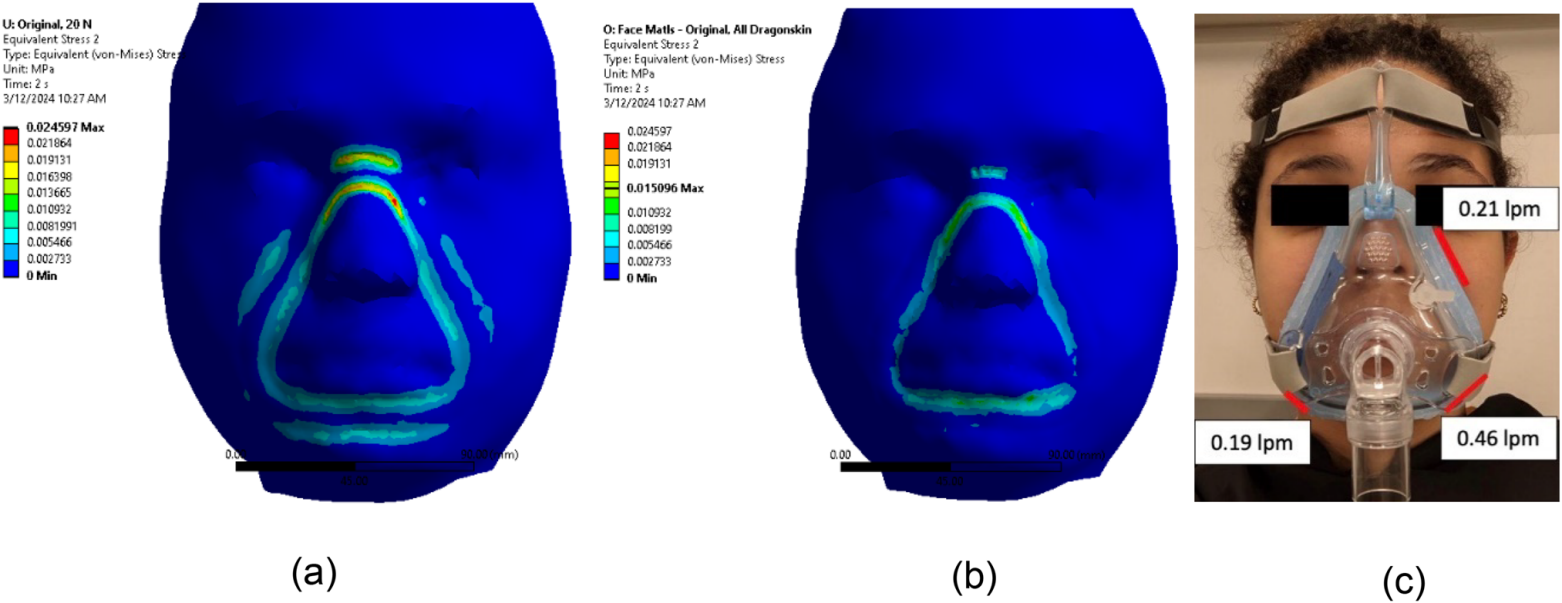
**a** Subject 1 stress results from previous model using arbitrary soft tissue thickness and elasticity values, **b** Subject 1 stress results with individual soft tissue thickness and elasticity values, and **c** in vivo leakage results for the original strap given in liters per minute (lpm)

We followed the same process described in our previous work to set up the simulation, except for the material assignments on the face. Only the 20 N load with the original strap was tested. Figure 10 shows the results from the previous model in comparison to the individualized model results as well as the in vivo leakage measured using a flow sensor. We immediately see that the stresses are much lower for the individualized model compared to the previous model. The maximum stress in the previous model is 24.60 kPa, while the maximum stress with the individualized model is 15.10 kPa. This is a 38% decrease in stress. In our prior work, we showed that reductions or gaps in the stress distribution could be regions of leakage. The stress results for the individualized model correlate better with the in vivo leakage regions compared to the previous model. The previous model has some reduction in stress at the regions where we observed in vivo leakage. However, with the individualized model, there are clear gaps in stress at the lower left and right corners of the mask and these correspond to leaks at those points. From these preliminary results, we see that adding individual soft tissue values results in a simulation that more closely represents real life. We also see that the individualized results are different compared to the results with arbitrary values. This highlights the importance of incorporating these individual measurements to properly assess the mask fit on a particular subject.

## Discussion

Measuring the material properties of human tissue in vivo is very valuable for producing accurate finite element models but collecting this information poses a significant challenge. The moduli of elasticity reported in Table 2 range from 7.53 to 117 kPa, showing the wide variation in elasticity of the facial tissues. However, these values are in line with other literature. Luboz et al. report a cheek stiffness of 31 ± 4.6 kPa and a cheekbone stiffness of 34.0 ± 6.6 kPa [10]. Our results were lower for the cheek with 16.20 ± 5.09 kPa and stiffer for the cheekbone with 64.79 ± 17.12 kPa, but they are still in the same order of magnitude as their results. Flynn et al. measured the elasticity of facial skin and reported cheek and forehead stiffnesses between 15.9 and 89.4 kPa [12]. Xu and Yang reviewed many sources that measured facial skin elasticity and found values ranging from 4.5 to 850 kPa [19]. Our results fall within these previously reported ranges of stiffness.

We found that the stiffnesses of all the sites tested were statistically different from one another except the cheek near lip and the cheek, as indicated in Table 3. Within each subject, the chin and cheekbone tended to be stiffer, while cheek and cheek near lip are less stiff. The below cheekbone site did not always follow this trend but was generally stiffer than the cheek and cheek near lip and less stiff than the chin and cheekbone. This shows the importance of assigning different material properties for different regions of the face to properly represent it in simulation. We also observed wide variation between subjects, as evidenced by the large standard deviation of the mean at each testing location. Based on the inter-subject variability, it is not sufficient to represent each face based on the average stiffnesses collected and it is necessary instead to use individual facial properties. This inter-subject variation emphasizes the need for an easy tool to characterize each subject’s facial soft tissue elasticity.

For thickness, the ultrasound used in this paper is the same type referenced in De Greef et al., who compiled a large-scale database of Caucasian facial soft tissue depth [16]. For female subjects with BMI 20 to 25 aged 18 to 29, they found the chin thickness as 9.6 ± 1.7 mm, cheekbone as 9.4 ± 2.1 mm, below cheekbone as 17.9 ± 2.7 mm, and the cheek as 19.4 ± 2.0 mm. For men of the same BMI and age they found the chin thickness as 9.5 ± 1.66 mm, cheekbone as 8.3 ± 2.07 mm, below cheekbone as 16.2 ± 2.8 mm, and the cheek as 25.0 ± 3.48 mm. The values we collected are slightly higher for the chin with 11.97 ± 2.08 mm, slightly lower for the cheekbone at 7.76 ± 2.02 mm, about the same for below cheekbone at 16.17 ± 4.22 mm, and about the same for the cheek with 26.67 ± 5.51 mm. Overall, the results we collected are in line with the results from De Greef et al. Other sources with different racial demographics report similar values for each of these sites [20–23].

When looking at facial soft tissue elasticity and thickness, we expect a negative correlation between stiffness and thickness, or that stiffness should increase as thickness decreases. The only testing location where this appeared to be the case was the cheekbone. However, the average elasticity and thickness did seem to show a possible correlation, although it is not currently statistically significant. Testing more subjects and more sites could reveal further correlations between elasticity and thickness.

There are various limitations to consider with the STM device. Firstly, we are modeling soft tissue as a homogeneous, isotropic, linear elastic material that has a constant elastic modulus along any indentation depth when in fact soft tissue is non-linear and heterogeneous. Regardless, we were able to validate the STM device against known elasticity ballistics gel samples, even though ballistics gel is also a non-linear elastic material [24]. The error for the validation of these samples did reach up to 40% for the softest sample, so we can expect a similar error from the facial tissue measurement. Ballistics gel is also homogeneous, while facial soft tissue is not; being made up of several layers of skin, muscle, and fat. For our application, we do expect the CPAP mask to indent quite far into the face, up to 5 mm or greater in softer areas like the cheek, cheek near lip, and below cheekbone. This means that the indentation will reach past the thickness of the skin and into the fat and muscle layers. The STM indents quite far into the tissue, and therefore captures the behavior of these subdermal tissues.

Secondly, there are many sources of human error. One factor that can strongly affect the stiffness results is muscle activation and the amount of resistance the subject places against the probe during testing. Since we are measuring soft tissue elasticity instead of just skin elasticity, muscle activation can greatly increase the stiffness. We ask subjects to keep their face relaxed during testing but cannot guarantee that there is no muscle activation. Some of the unexpectedly stiff measurements could be explained by muscle activation such as the chin measurement of 117.00 kPa for Subject 18 or the cheek near lip measurement of 41.90 kPa for Subject 17. Subjects are also asked to resist the force of the probe as it indents their face, and the amount of resistance they provide may be variable. This could be mitigated by including a strap around the back of the subject’s head to keep it in place during testing. Another source of human error is the manual placement of the probe at each site. We visually identify the position of the probe perpendicular to the surface of the face and just touching the face, which can lead to error. Despite this, the device appears to have good precision, with the standard deviation being on average 10% of the mean of the five samples tested per facial site.

Finally, a third limitation is in our sample group. Most of the subjects tested were young, with 26 subjects aged 19–32 and just two aged 54. Many patients who use CPAP are older than the age group tested so more tests would be needed to evaluate the STM on older people. Subjects also mostly had normal BMI which is not representative of the United States population. Finally, the demographic breakdown of the subjects is also not representative of the United States. Testing the device on a greater age and BMI range may reveal more correlations between facial soft tissue elasticity and these metrics.

We also completed an initial FE simulation for one subject, incorporating some soft tissue thickness and elasticity measurements. The facial soft tissue measurements were collected for Subject 1 from a previous FE paper and the results were compared to the previous results with arbitrary soft tissue values. The initial results showed that the individualized FE model resulted in lower stress and a different stress distribution compared to the previous model. The individualized model stress results also correlated more closely with in vivo leakage results. This initial attempt serves as a proof of concept on how to incorporate the soft tissue measurements into FE simulations. However, more subjects need to be tested to understand the impact of incorporating these measurements on the results. We could also incorporate more of the soft tissue measurements into the FE model.

In summary, this paper presents a novel indenter device to estimate facial soft tissue elasticity. It is affordable and easy to build with mostly off-the-shelf parts, and only a few custom additively manufactured pieces. This makes the device more accessible for other researchers to recreate compared to the highly engineered devices and methods developed by others. The STM device was validated on ballistics gel samples and subsequently tested on 28 subjects, whose facial tissue thickness was also measured using ultrasound. Elasticity and thickness results were in the correct range of values compared to other work. The STM device has good precision, but more work still needs to be done to validate the accuracy and understand further trends between elasticity and thickness. Despite this, we have validated the use of our STM device to collect facial soft tissue thicknesses and elasticities for use in FE simulations to evaluate CPAP mask fit and showed the results of an initial simulation incorporating facial soft tissue measurements.
